# Hepcidin as a Diagnostic Biomarker in Anaemic Lung Cancer Patients

**DOI:** 10.3390/cancers15010224

**Published:** 2022-12-30

**Authors:** Katarzyna Wadowska, Piotr Błasiak, Adam Rzechonek, Iwona Bil-Lula, Mariola Śliwińska-Mossoń

**Affiliations:** 1 Department of Medical Laboratory Diagnostics, Division of Clinical Chemistry and Laboratory Haematology, Faculty of Pharmacy, Wroclaw Medical University, Borowska 211A, 50-556 Wroclaw, Poland; 2 Department and Clinic of Thoracic Surgery, Faculty of Medicine, Wroclaw Medical University, Grabiszyńska 105, 53-439 Wroclaw, Poland; 3 Lower Silesian Centre of Oncology, Pulmonology and Haematology, Lower Silesian Thoracic Surgery Centre, Grabiszyńska 105, 53-439 Wroclaw, Poland

**Keywords:** lung cancer, non-small cell lung carcinoma, anaemia of chronic disease, anaemia of inflammation, iron deficiency anaemia, biomarkers, hepcidin, overall survival, quality of life

## Abstract

**Simple Summary:**

Lung cancer is the leading cause of cancer-related deaths, with a low overall survival rate. Anaemia is one of the most common cancer side effects, reducing the patient’s survival time among other things. Lung cancer patients have the highest incidence of anaemia, with 50–70% of patients experiencing anaemia during the course of their disease. In clinical practice, however, anaemia is not a problem in cancer patients until it becomes severe or life-threatening, at which point therapeutic actions are taken. We choose to change this perception by first investigating the characteristics of proteins involved in the pathogenesis of anaemia, as well as their diagnostic capabilities. We assume that predicting anaemia using diagnostic biomarkers, and thus preventing and treating anaemia in lung cancer patients, is beneficial to both the patient and the economy.

**Abstract:**

We aim to describe the characteristics of hepcidin, IL-6, and TNF-α levels in anaemia of lung cancer patients with operative tumour as well as to investigate the potential diagnostic capabilities of hepcidin in combination with IL-6, TNF-α, and acute phase proteins. We present a retrospective study of 112 lung cancer patients (41 women and 71 men) who were surgically treated at the Lower Silesian Centre for Lung Diseases in Wroclaw, Poland. Serum blood samples were collected from all these patients prior to any surgical treatment and used to determine hepcidin, IL-6, TNF-α, SAA_1_, and CRP concentrations. Patients were also examined with a complete blood count several times during their hospitalization. The female and male groups were divided based on the occurrence of anaemia during their hospitalization. Patients who developed anaemia post-operatively had significantly lower hepcidin concentrations than non-anaemic patients (*p* = 0.000694 in females with ≥3 complete blood count examinations and *p* = 0.007905 in males with 2 complete blood count examinations), whereas patients with anaemia since hospital admission had higher hepcidin concentrations. We observed two hepcidin roles related to two cancer anaemia pathogeneses: (1) higher hepcidin concentrations in patients with anaemia since hospital admission (anaemia of inflammation) and (2) lower hepcidin concentrations in patients who developed anaemia after surgery (anaemia of iron deficiency). Our data support the role of hepcidin, IL-6, and TNF-α in cancer-related anaemia and provide diagnostic values for predicting post-operative anaemia in lung cancer patients.

## 1. Introduction

Lung cancer is the most common cause of cancer death, accounting for nearly one-quarter of all cancer deaths. It is estimated that lung cancer will cause more deaths than breast, prostate, and colon cancer together [1,2].

The vast majority of patients with lung cancer (75%) are diagnosed at an advanced stage of the disease, when treatment options are limited, contributing to the high mortality rate. Even as lung cancer treatments and outcomes improve, survival still remains a challenge. Overall five-year relative survival rates increased only slightly over the last 50 years, from 12.4% for 1974–1976 diagnoses to 15.0% for 1996–2002 diagnoses and to 22% in 2020 for all people with all types of lung cancer. Survival rates differ depending on factors such as the subtype of lung cancer, the stage of the disease, and even the gender of the patient [3,4,5].

Anaemia is one of the most common side effects of both blood cancers and solid tumours, and it has far-reaching consequences for the entire body and cancer treatment, including reduced survival time and sensitivity to chemotherapy and radiotherapy. According to the findings of The European Cancer Anaemia Survey (ECAS), approximately 40% of patients with solid tumours involve anaemia. Among solid tumour patients, patients with lung cancer have the highest incidence of anaemia: approximately 50–70% of patients with lung cancer experience anaemia during the course of their disease [6,7,8,9,10,11,12].

Haemoglobin levels are used in clinical practice to define anaemia and its severity, according to World Health Organisation (WHO) and National Cancer Institute (NCI) classification/grading, among others (Table 1). However, the assessment of anaemia’s severity is of limited value because it does not allow for an evaluation of anaemia’s nature, morphological type, or mechanisms underlying its development [6,7,13,14]. Anaemia of cancer is also known as anaemia of chronic disease (ACD) or anaemia of inflammation (AI), and it has a multifactorial aetiology that includes cancer progression, coexisting inflammation, renal or bone marrow involvement, malnutrition, and oncologic treatment such as chemotherapy and radiotherapy [13,14,15].

The pathophysiology of ACD/AI involves immune activation in response to tumour antigens with release of several pro-inflammatory cytokines by immune cells, including interleukin (IL)-1, IL-6, IL-22, tumour necrosis factor (TNF)-α, interferon (IFN)-γ, and transforming growth factor (TGF)-β which stimulate hepatic synthesis of hepcidin. Hepcidin is a circulating hormone of inflammation that is the primary regulator of iron availability to developing red blood cells (RBCs) by inhibiting iron release from macrophages and interfering with intestinal iron absorption. Moreover, the pathophysiology of ACD/AI involves immune-mediated effects on the proliferation of erythroid progenitor cells, red blood cell turnover and half-life, and biological activity of erythropoietin. [6,10,12,16,17,18].

The objective of this study was to describe the characteristics of hepcidin, IL-6, and TNF-α levels in anaemia of lung cancer patients with operative tumours. We also wanted to investigate the potential diagnostic capabilities of hepcidin in combination with IL-6, TNF-α, and acute phase proteins such as C-reactive protein (CRP) and serum amyloid A_1_ (SAA_1_), via laboratory assessment of the anaemia’s basis and prediction of the occurrence of post-operative anaemia.

## 2. Materials and Methods

### 2.1. Patients

A total of 112 lung cancer patients were included in the study. All participants were recruited by the Department of Thoracic Surgery, Lower Silesian Centre for Lung Diseases in Wroclaw, Poland, and signed a written informed consent following an explanation of the study protocols. The study protocol conformed to the World Medical Association’s Declaration of Helsinki (2000) and was approved by the Bioethics Committee at the Wroclaw Medical University (NR KB: 106/2020).

All patients were surgically treated and underwent lobectomy (61 out of 112, 54.46%), wedge resection (28 out of 112, 25.00%), biopsy (9 out of 112, 8.04%), segmentectomy (7 out of 112, 6.25%), bilobectomy (5 out of 112, 4.46%), or pulmonectomy (2 out of 112, 1.79%). Diagnosis of lung cancer was confirmed by histopathological examination, performed on the tumour tissue obtained. The lung cancer diagnosis was established in accordance with the National Comprehensive Cancer Network (NCCN) Clinical Practice Guidelines in Oncology and was staged in accordance with the American Joint Committee on Cancer’s (AJCC) 8th TNM Staging System. Clinical, laboratory, and pathological data for these patients were acquired from hospital medical records using the AMMS IT system (Asseco Medical Management Solutions). The essential characteristics of patients for this study, divided by gender, are presented in Table 2.

### 2.2. Methods

#### 2.2.1. Study Design

Prior to any surgical treatment, venous blood samples were collected into tubes with clot activator from all patients. Blood samples were centrifuged at 2000× *g* for 8–10 min at room temperature to separate sera, which were then stored at −80 °C until use. Serum blood samples were used to determine the concentrations of IL-6, TNF-α, SAA_1_, hepcidin, and CRP. Commercial enzyme-linked immunosorbent assays (ELISA) test kits: Human IL-6 DuoSet ELISA (Catalog # DY206), Human TNF-alpha DuoSet ELISA (Catalog # DY210), Human Serum Amyloid A1 DuoSet ELISA (Catalog # DY3019-05), and Human Hepcidin DuoSet ELISA (Catalog # DY8307-05), R&D Systems, Inc., Minnesota, MN, USA, were used in accordance with manufacturer’s protocols. The detection limits of the used ELISA kits correspond to the standard curves, i.e., Human IL-6 DuoSet ELISA: 9.38–600 pg/mL, Human TNF-alpha DuoSet ELISA: 15.63–1000 pg/mL, Human Serum Amyloid A1 DuoSet ELISA: 1.56–100 ng/mL (we performed 1000-, 2000-, and 3000-fold reconstitution using serial dilutions), Human Hepcidin DuoSet ELISA: 3.13–200 pg/mL (we performed 1000-, 2000-, and 3000-fold reconstitution using serial dilutions). The sensitivity of our ELISA kits, also known as the Lowest Limit of Detection (LLOD), is the lowest calculated/statistical point that is different from zero that the kit can statistically detect. We performed the analysis in the double-check mode, which means we measured each sample twice to reduce the likelihood of random errors. CRP levels were determined using a commercial kit based on turbidimetric method: C-REACTIVE PROTEIN (Catalog # 31321), BioSystems S.A., Barcelona, Spain, in accordance with the manufacturer’s instructions. The detection limits for CRP determination are 1.0–150 mg/L, the repeatability CVs (in series) are 3.6–4.5%, and the reproducibility CVs (between series) are 3.7–4.6%.

Moreover, venous blood samples were collected several times during patients’ hospitalization into tubes containing ethylenediaminetetraacetic acid (EDTA): before surgery, on the day of surgery (after surgery), and at longer intervals following surgery to determine peripheral blood count. Complete blood counts (CBCs) were analysed in the hospital’s clinical laboratory using automated haematology analysers XN-550 and XN-1000 (Sysmex Corporation, Kobe, Japan).

#### 2.2.2. Grouping Methods

The research group was divided based on gender due to gender-related differences in haemoglobin concentration. Then, the women (*n* = 41) and men (*n* = 71) were both divided into three groups based on the number of CBCs performed during operable patients’ hospitalization. A total of 14 women and 26 men were examined ≥3 times during follow-up: before surgery, on the day of surgery after surgery, and in the days following surgery. During their hospitalization, 23 women were examined twice, once before surgery and once just after surgery, whereas 4 women were examined only once, upon admission to the hospital. In the case of men, 38 were examined twice during their hospitalization, while 7 were examined only once, including 6 patients who were examined only upon admission to the hospital and 1 patient who was examined only shortly after surgery.

Then, we analysed peripheral blood count parameters from CBC results obtained from patients upon admission to the hospital, post-operatively, and in the days following surgery. We chose the result with the lowest haemoglobin concentration from patients who had ≥3 CBC examinations and applied parameters from these results to the analyses as the third examination. We also considered the occurrence of anaemia at all stages of the patient’s hospitalization. Using haemoglobin levels less than 11 g/dL as the diagnostic standard for anaemia in women and 13.5 g/dL for men, we grouped patients into anaemia groups (including anaemia since the day of hospital admission and anaemia developed during the course of the hospitalization) and non-anaemia groups. We reduced the risk of incorrectly classifying patients who might have developed anaemia during their hospitalization as non-anaemia patients by dividing the study group based on the number of CBCs performed. Patients with anaemia since the day of hospital admission, as well as those with CBC examined three or more times throughout their hospitalization, i.e., with an evident and specified endpoint, provided us with the most accurate and valuable information. Figures S1 and S2 in the Supplement Materials show how the study group was divided and how the incidence of anaemia was distributed.

### 2.3. Statistical Analysis

The obtained data were statistically analysed using TIBCO Software Inc. (Palo Alto, CA, USA) (2017), Statistica, version 13 (http://statistica.io, accessed on 16 December 2021 and 27 June 2022) with the additional Plus Package (version 5.0.96), and a significance level of *p* < 0.05. The Shapiro–Wilk test was used to determine whether the data for each parameter was normally distributed across all analysed groups. We used chi-square test to compare discrete variables. The parametric Student’s *t*-test and one-way analysis of variance (ANOVA) were used to compare independent, continuous variables between two and more groups, respectively. Repeated measures ANOVA was used to compare parameters from consecutive CBCs examinations. Post hoc analyses using the Student–Newman–Keuls method and its modification, Duncan’s new multiple range test, supplemented ANOVA. We used the Pearson correlation in variates method to compute the pairwise correlations between CBC parameters from consecutive examinations and analysed protein concentrations in women and men based on the number of CBC examinations and the presence of anaemia.

## 3. Results

### 3.1. The Incidence of Anaemia in the Consecutive Stages of Hospitalization

**Figure 1:**
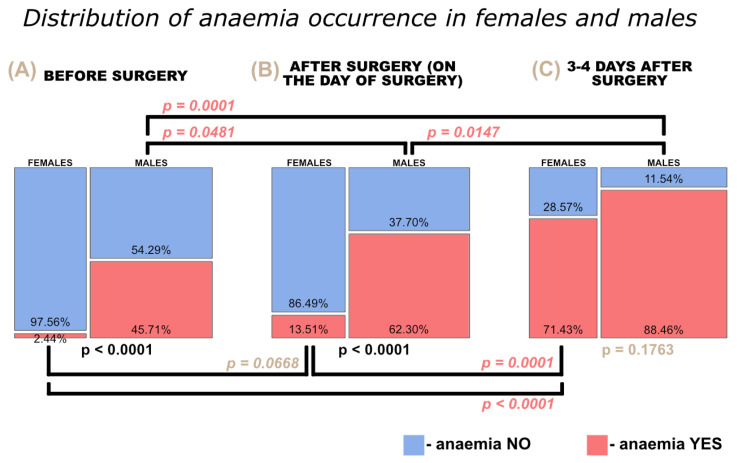
Comparison of anaemia prevalence in women and men (**A**) before surgery, (**B**) after on the day of surgery, and (**C**) 3–4 days after surgery.

The study included 41 women (36.61%) and 71 men (63.39%). Using haemoglobin levels less than 11 g/dL as the diagnostic standard for anaemia in women, we found only 1 (2.44%) patient with anaemia and 40 (97.66%) patients without anaemia upon admission to the hospital. Among these 40 patients, 12 (29.27%) patients developed anaemia during their hospitalization and 28 (68.29%) did not. Using haemoglobin levels less than 13.5 g/dL as the diagnostic standard for anaemia in men, we discovered that nearly half of the male lung cancer group (32 out of 71, 45.07%) had anaemia upon admission to the hospital. Moreover, another 16 (22.54%) patients developed anaemia post-operatively, while 23 (32.39%) had no anaemia during their hospitalization. Figure 1 shows the prevalence of anaemia in women and men in the consecutive stages of hospitalization, i.e., upon admission to the hospital, on the day of surgery after surgery, and in the days following surgery, as well as a comparison of its prevalence by gender.

We used contingency tables to summarize the relationships between the gender of the patients and the prevalence of anaemia during the course of their hospitalization. Using the chi-square test, we found statistically significant differences in the prevalence of anaemia between females and males before surgery (*p* < 0.0001) and after surgery (on the day of surgery, *p* < 0.0001), with males having a higher frequency of anaemia. We also noticed a statistically significant increase in the frequency of anaemia in both females and males over the course of their hospitalization. In the case of women, we found a significant increase in anaemia prevalence between hospital admission and 3–4 days after surgery (*p* < 0.0001), as well as between the day of surgery (after surgery) and 3–4 days after surgery (*p* = 0.0001). We observed a significant increase in anaemia prevalence in men between the day of admission to hospital and the day of surgery (after surgery) (*p* = 0.0481), as well as between the day of admission to hospital and 3–4 days after surgery (*p* = 0.0001), and between the day of surgery (after surgery) and 3–4 days after surgery (*p* = 0.0147) (Figure 1).

### 3.2. Complete Blood Count Parameters

The incidence of anaemia in women and men was reflected in the levels of CBC parameters. In women, we found haemoglobin concentrations and red blood cell counts within the laboratory’s reference ranges in the first two stages of hospitalization. Normal haemoglobin concentrations (13.15 g/dL before, and 12.44 g/dL after) and red blood cell counts (4.37 × 10^6^/μL before, and 4.13 × 10^6^/μL after) before and after surgery (on the day of surgery) are related to a low prevalence of anaemia in women, with 2.44% and 13.51%, respectively. Only in a CBC performed 3–4 days after surgery we found a decrease in haemoglobin concentration (10.13 g/dL), as well as haematocrit (30.71%) and red blood cell counts (3.42 × 10^6^/μL) below the laboratory’s reference values, indicating anaemia in women.

Since the first stage of the patient’s hospitalization, the mean haemoglobin concentrations and red blood cell count values in men have been lower than the laboratory’s reference values, decreasing from 13.39 g/dL to 10.99 g/dL in haemoglobin concentration and from 4.44 × 10^6^/μL to 3.69 × 10^6^/μL in red blood cell count values. The mean corpuscular volume (MCV), mean corpuscular haemoglobin (MCH), and mean corpuscular haemoglobin concentration (MCHC) of red blood cells did not deviate from the laboratory’s references values in either women or men, indicating normocytic normochromic anaemia. Table S1 in the supplement materials shows the values of CBC parameters at each stage of the patient’s hospitalization in relation to the laboratory’s reference values.

### 3.3. The Effect of Independent Variables (Subtype and Stage of Lung Cancer etc.) on the Values of Complete Blood Count Parameters

Prior to examining the influence of hepcidin, IL-6, and TNF-α on CBC parameters, we investigated the potential impact of subtype and stage of lung cancer, type and extent of surgery that patient underwent, presence of inflammation in the tumour environment (observed in the tumour histopathological examination as inflammatory infiltration next to dust deposition and necrosis), patients’ smoking status, and underlying lung diseases and other comorbidities on the values of CBC parameters in both women and men. We found statistically significant differences in haemoglobin concentrations and haematocrit values before surgery between lung cancer subtypes in women. A post hoc analysis did not reveal which lung cancer subtypes had statistically significant differences in haematocrit values. However, we found that haemoglobin concentrations differ between lung adenocarcinoma ($\bar{x}=$ 13.8 g/dL ± 0.3) and lung squamous cell carcinoma ($\bar{x}=$ 12.3 g/dL ± 0.3; *p* = 0.039425). In women with adenocarcinoma, the haematocrit value was $\bar{x}=40.0\%$, and in squamous cell carcinoma, it was $\bar{x}=37.5\%$. The mean haemoglobin concentrations and haematocrit values in both cases of adenocarcinoma and squamous cell carcinoma patients were fitted in the laboratory’s reference values and did not indicate anaemia in these patients.

In the case of the remaining analysed variables, there were no statistically significant differences in the values of CBC parameters in both women and men. We concluded that the stage of lung cancer, the type and extent of surgery that patients underwent, the presence of inflammation in the tumour environment, and the patient’s smoking status had no effect on the values of CBC parameters.

### 3.4. Analysis of Hepcidin, IL-6, and TNF-α Concentrations in Females

We began the analysis by examining the effect of independent variables (subtype and stage of lung cancer, etc.), on the levels of hepcidin, IL-6, and TNF-α. There were no statistically significant differences in hepcidin, IL-6, and TNF-α concentrations between lung cancer subtypes or stages, as well as between patients with and without underlying lung diseases and other comorbidities such as type 2 diabetes mellitus and hypertension in female patients.

Then, we compared the group of females divided based on the number of CBCs performed, and we observed statistically significant differences in the number of hospitalization days between patients who had one ($\bar{x}=5.5\;\mathrm{days}$) or ≥3 ($\bar{x}=9.7\;\mathrm{days}$) (*p* = 0.002569) CBC examinations, as well as between patients who had two ($\bar{x}=6.7\;\mathrm{days}$) or ≥3 ($\bar{x}=9.7\;\mathrm{days}$) (*p* = 0.000361) CBC examinations. Women who had three or more CBC verifications spent 3 to 4 days longer in the hospital than patients who were only examined once or twice during their hospitalization.

#### 3.4.1. A Group of Women with One Complete Blood Count

A CBC was only performed once on four women, upon admission to the hospital. One of the patients was anaemic (haemoglobin concentration = 10.5 g/dL), while the other three were not. The anaemia patient in this group was the only one of the 41 lung cancer women in the study group who had anaemia prior to surgery, which could be attributed to the patient’s advanced lung cancer at stage IVA, because of oesophageal squamous cell carcinoma metastases in the respiratory tract. Anaemia patients had higher cytokine concentrations (126.81 pg/mL vs. 8.74 pg/mL for IL-6 and 302.38 pg/mL vs. 2.09 pg/mL for TNF-α) and more than double the increase in the hepcidin concentration than non-anaemia patients in this group (194.56 ng/mL and $\bar{x}=80.02\;\mathrm{ng}/\mathrm{mL}$, respectively), but lower CRP and SAA_1_ concentrations (2.78 mg/L vs. 9.57 mg/L for CRP and 81.11 μg/mL vs. 131.07 μg/mL for SAA_1_). Descriptive statistics for all studied proteins are also included in Table S2 in the supplemental materials.

Moreover, we found a positive, strong, statistically significant correlation between IL-6 and hepcidin (r = 0.99, *p* = 0.045) and IL-6 and TNF-α concentrations (r = 0.99, *p* = 0.026) in a group of women with one CBC. All correlations are listed in Table 3.

#### 3.4.2. A Group of Women with Two Complete Blood Counts

A CBC was performed on 23 women twice during their hospitalization, once upon admission and once on the day of surgery (after surgery). Only two women in this group had post-operative anaemia. Statistical analyses revealed no significant differences in the concentrations of analysed proteins in this group of patients. However, patients with post-operative anaemia had lower mean hepcidin concentration ($\bar{x}=49.75\;\mathrm{ng}/\mathrm{mL}$) and higher mean IL-6 concentration ($\bar{x}=31.54\;\mathrm{pg}/\mathrm{mL}$) than non-anaemia patients ($\bar{x}=106.33\;\mathrm{ng}/\mathrm{mL}$ and $\bar{x}=26.42\;\mathrm{pg}/\mathrm{mL}$, respectively). Table S3 in the supplementary materials provides descriptive statistics for all studied proteins.

However, we found a positive, statistically significant correlation between TNF-α and SAA_1_ concentrations (r = 0.55, *p* = 0.013), as well as between CRP concentration and the number of days patients were hospitalized (r = 0.57, *p* = 0.008). Table 3 contains a list of all correlations.

#### 3.4.3. A Group of Women with ≥3 Complete Blood Counts

On admission to the hospital, no patient in the group with ≥3 CBCs had anaemia, whereas only four women were non-anaemic in the third CBC examination.

**Figure 2:**
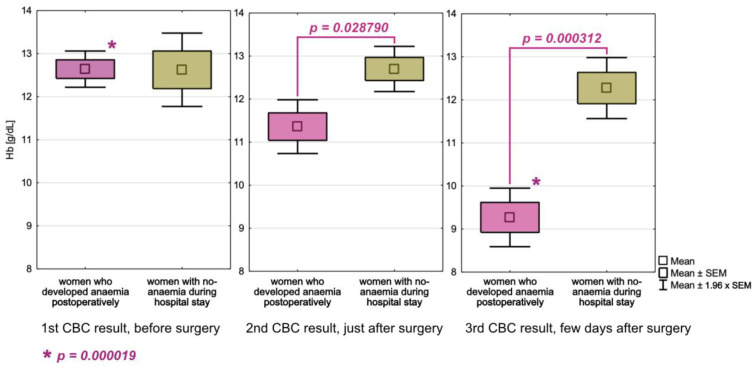
Dynamics of haemoglobin level changes in three consecutive complete blood count examinations in anaemia and non-anaemia women from a group of women with ≥3 complete blood counts.

When comparing haemoglobin concentrations upon admission to the hospital between women that developed anaemia in the course of hospitalization and non-anaemia women, there were practically no differences in the means, with values of 12.64 g/dL and 12.63 g/dL, respectively. However, we observed a significant decrease in haemoglobin concentration in anaemia patients (from 12.64 g/dL to 9.27 g/dL, *p* = 0.000019) and a slight decrease in non-anaemia patients (from 12.63 g/dL to 12.28 g/dL, *p* = 0.188120) during hospitalization. Furthermore, when we compared haemoglobin concentrations in anaemia and non-anaemia women in consecutive CBC examinations, we found statistically significant differences in the second ($\bar{x}=11.36$ g/dL and $\bar{x}=12.70$ g/dL, *p* = 0.028790), and third ($\bar{x}=9.27$ g/dL and $\bar{x}=12.28$ g/dL, *p* = 0.000312) CBC examination. The dynamics of haemoglobin level changes in anaemia and non-anaemia women in consecutive CBC examinations are depicted in Figure 2. Descriptive statistics for CBC parameters are also included in Table 4.

**Figure 3:**
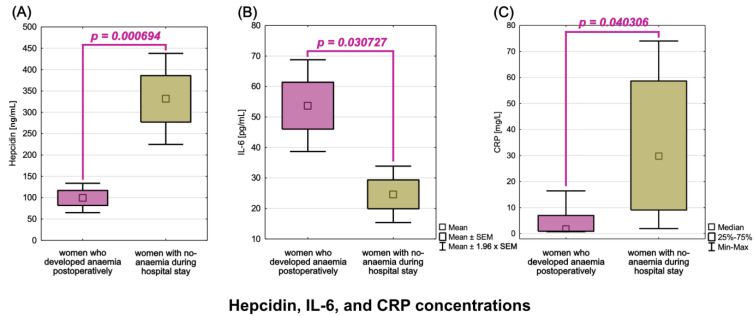
Hepcidin (**A**), IL-6 (**B**), and CRP (**C**) levels in women with and without post-operative anaemia.

Furthermore, when we compared hepcidin and cytokines levels in anaemia and non-anaemia women, we discovered statistically significant differences in hepcidin and IL-6 concentrations. Anaemia patients had lower hepcidin concentration ($\bar{x}=99.34$ ng/mL) than non-anaemia patients ($\bar{x}=331.43$ ng/mL, *p* = 0.000694), with simultaneously higher IL-6 levels ($\bar{x}=53.72$ pg/mL) than non-anaemia patients ($\bar{x}=24.64$ pg/mL, *p* = 0.030727). The power analysis of the obtained results revealed that the hepcidin analysis had a high power of 0.86 and the IL-6 analysis had a power of 0.51. Moreover, we discovered statistically significant differences in CRP concentrations, with non-anaemia women having significantly higher CRP levels (me = 29.74 mg/L) than women who developed anaemia post-operatively (me = 1.85 mg/L, *p* = 0.040306). Figure 3 depicts the differences in hepcidin, IL-6, and CRP concentrations in women with and without post-operative anaemia. Table 4 also includes descriptive statistics for all analysed proteins with marked statistical significance.

We also performed correlation analyses. In a group of women who had ≥3 CBCs, we found a strong, statistically significant, positive correlation between CRP and SAA_1_ concentrations (r = 0.92, *p* = 0.000). Furthermore, we observed a statistically significant negative correlation between hepcidin concentration and RBC value (r = −0.7531, *p* = 0.019) from the first CBC, as well as statistically significant positive correlations between hepcidin concentration and haemoglobin concentration (r = 0.7282, *p* = 0.026), haematocrit (r = 0.7189, *p* = 0.029), and RBC values (r = 0.6986, *p* = 0.036) from the third CBC. All correlations are listed in Table 3.

### 3.5. Analysis of Hepcidin, IL-6, and TNF-α Concentrations in Males

**Figure 4:**
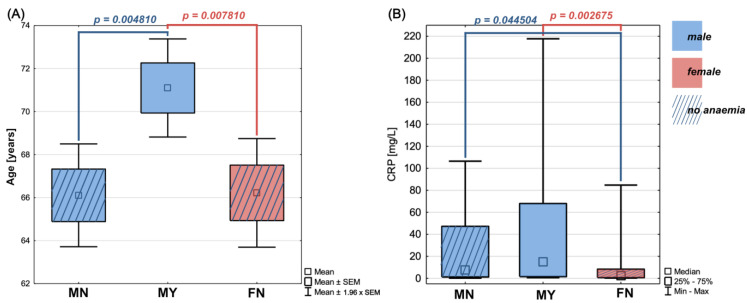
Age (**A**) and CRP levels (**B**) disparities between patients with and without pre-operative anaemia; MN—males without anaemia; MY—males with anaemia; FN—females without anaemia.

Almost half of the men in our study group had anaemia when they were admitted to the hospital. We first looked to see if there were any differences in the essential characteristics of anaemia patients and no anaemia patients, such as subtype and stage of lung cancer, presence of inflammation in the tumour environment, patients’ smoking status, or age. The characteristics of males admitted to the hospital with anaemia did not differ from those of the overall group of male patients in our studied lung cancer population (Table 2). There were, however, age differences between males with anaemia and the rest of our study group. We discovered statistically significant age differences between anaemia and non-anaemia males (*p* = 0.004810), as well as anaemia males and non-anaemia females (*p* = 0.007810), with anaemia males being 5 years older on average than non-anaemia males and non-anaemia females. Because of the small number of patients (N = 1), we did not include the group of females with pre-operative anaemia in the analysis. Figure 4 depicts the age disparities between patients with and without pre-operative anaemia, demonstrating that females and males without pre-operative anaemia are of comparable age, in contrast to anaemia males.

Then, we looked to see if there were any statistically significant differences in protein concentrations between groups based on the presence of anaemia at the time of admission to the hospital. We found that non-anaemia women had significantly lower hepcidin concentrations ($\bar{x}=125.27\;\mathrm{ng}/\mathrm{mL}$) than non-anaemia men ($\bar{x}=174.64\;\mathrm{ng}/\mathrm{mL}$, *p* = 0.041324). Simultaneously, we discovered that non-anaemia women had significantly lower CRP concentrations (me = 2.27 mg/L) than non-anaemia men (me = 7.56 mg/L, *p* = 0.044504) and anaemia men (me = 15.06 mg/L, *p* = 0.002675). Table 5 provides descriptive statistics for age and analysed proteins in women and men, subdivided by the presence of anaemia at the time of admission to the hospital.

We also examined the effect of independent variables (subtype and stage of lung cancer etc.) on hepcidin, IL-6, and TNF-α concentrations in male lung cancer patients. There were no statistically significant differences in hepcidin, IL-6, and TNF-α concentrations between lung cancer subtypes or stages, or between patients with and without underlying lung diseases. However, we found statistically significant differences in IL-6 concentrations between males with anaemia since hospital admission and comorbidities and those without comorbidities. Anaemic patients with comorbidities (type 2 diabetes mellitus, hypertension, chronic obstructive pulmonary disease) had significantly higher IL-6 concentrations ($\bar{x}=46.33\;\mathrm{pg}/\mathrm{mL}$) than anaemic patients without comorbidities ($\bar{x}=21.92$ pg/mL) (*p* = 0.005271). There were no such differences in men who developed anaemia during hospitalization, and in men who did not develop anaemia during hospitalization. Table S4 of the Supplementary Materials contains all descriptive statistics for the proteins studied.

Finally, we compared a group of males divided by the number of CBCs performed and found statistically significant differences in the number of hospitalization days between patients who had two ($\bar{x}=7.0\;\mathrm{days}$) or ≥3 ($\bar{x}=9.0\;\mathrm{days}$) (*p* = 0.001500) CBC examinations. Men who had three or more CBC verifications spent two to three days longer in the hospital than patients who were only examined once or twice during their hospitalization.

#### 3.5.1. A Group of Men with One Complete Blood Count

A CBC was performed only once on seven men, five of whom were non-anaemic and two of whom had anaemia upon admission to the hospital. In this group, there were no statistically significant differences in hepcidin, IL-6, or TNF-α levels between anaemia and non-anaemia men. However, compared to non-anaemic men, men with anaemia had hepcidin concentrations that were twice as low ($\bar{x}$ = 155.97 ng/mL vs. $\bar{x}$ = 82.09 ng/mL, respectively), and IL-6 concentrations that were marginally higher ($\bar{x}$ = 12.91 pg/mL vs. $\bar{x}$ = 15.59 pg/mL, respectively). All descriptive statistics of analysed proteins are included in Table S5 of the Supplementary Materials.

We also performed correlation analyses and discovered a strong negative, statistically significant relationship between TNF-α concentration and the number of red blood cells (r = −0.99, *p* = 0.014), as well as between CRP and haemoglobin concentrations (r = −0.96, *p* = 0.035). We also found a strong negative correlation (r = −0.90) between IL-6 and TNF-α concentrations, but it was not statistically significant (*p* = 0.098). Table 3 contains a list of all statistically important correlations.

#### 3.5.2. A Group of Men with Two Complete Blood Counts

**Figure 5:**
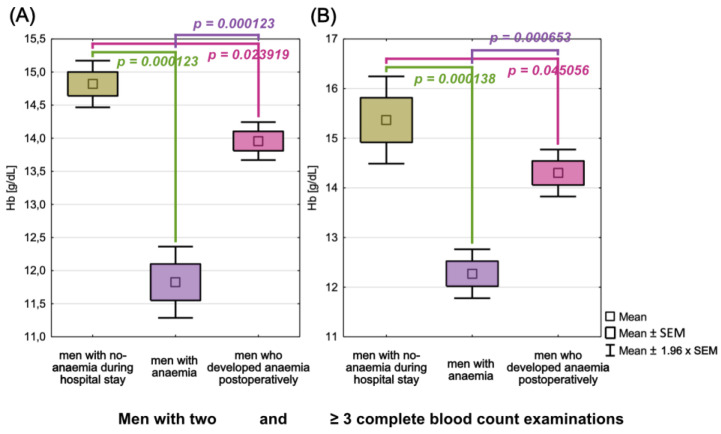
Differences in haemoglobin concentrations in the first complete blood count examination between men with and without anaemia, as well as men who developed anaemia post-operatively, who had two (**A**) and ≥ 3 complete blood count examinations (**B**).

A CBC was performed twice on 38 men, 15 of whom had no anaemia during their hospitalization, 16 had anaemia since their admission, and 7 developed anaemia while in the hospital. When we compared haemoglobin concentrations upon admission to the hospital between men who developed anaemia during their hospitalization, non-anaemia patients, and anaemia patients, we discovered statistically significant differences in each group, i.e., between men who developed anaemia and non-anaemia patients (*p* = 0.023919), anaemia and non-anaemia patients (*p* = 0.000123), and men who developed anaemia and anaemia patients (*p* = 0.000123). The highest haemoglobin concentrations were found in non-anaemia men ($\bar{x}=14.82$ g/dL), slightly lower in men who developed anaemia ($\bar{x}=13.96$ g/dL), and the lowest in anaemia patients ($\bar{x}=11.83$ g/dL). Figure 5 depicts differences in haemoglobin concentrations from the first CBC examination between groups.

**Figure 6:**
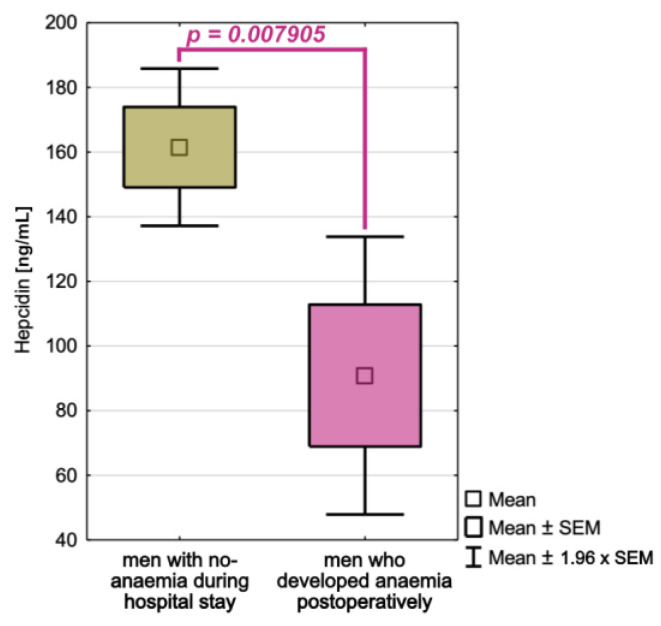
Hepcidin concentration differences between men without anaemia and men who developed anaemia during hospitalization and had two complete blood count examinations.

We found statistically significant differences in hepcidin concentrations between non-anaemia men and men who developed anaemia when we compared hepcidin, IL-6, and TNF-α concentrations between non-anaemia and anaemia patients, as well as men who developed anaemia during their hospitalization. Men with developed anaemia had significantly lower hepcidin concentrations (x = 90.84 ng/mL) than non-anaemia patients (x =161.50 ng/mL, *p* = 0.007905). The obtained results’ power analysis revealed that the hepcidin analysis in this group had a satisfactory (as for medical research) power of 0.66. Figure 6 depicts differences in hepcidin concentrations between non-anaemia men and men who developed anaemia during their hospitalization. Table S6 of the Supplementary Materials also includes descriptive statistics for all analysed proteins.

We also performed correlation analyses and discovered a positive, statistically significant relationship between CRP and SAA_1_ concentrations (r = 0.51, *p* = 0.018). Table 3 contains a list of all statistically important correlations.

#### 3.5.3. A Group of Men with ≥3 Complete Blood Counts

A CBC was performed thrice on 26 men, 3 of whom had no anaemia during their hospitalization, 14 had anaemia since their admission, and 9 developed anaemia while in the hospital. When we compared haemoglobin concentrations upon admission to the hospital between men who developed anaemia during their hospitalization, non-anaemia patients, and anaemia patients, we discovered statistically significant differences in each group, i.e., between men who developed anaemia and non-anaemia patients (*p* = 0.045056), anaemia and non-anaemia patients (*p* = 0.000138), and men who developed anaemia and anaemia patients (*p* = 0.000653). The highest haemoglobin concentrations were found in non-anaemia men ($\bar{x}=15.37$ g/dL), slightly lower in men who developed anaemia ($\bar{x}=14.30$ g/dL), and the lowest in anaemia patients ($\bar{x}=12.27$ g/dL). Figure 6 depicts differences in haemoglobin concentrations from the first CBC examination between groups. Table 6 includes descriptive statistics for CBC parameters (haemoglobin concentration and RBC) from both the first and third examinations.

We found no statistically significant differences in hepcidin, IL-6, TNF-α, CRP, and SAA_1_ concentrations between non-anaemia and anaemia patients, as well as men who developed anaemia during their hospitalization. Table 6 compiles descriptive statistics for hepcidin, IL-6, TNF-α, CRP, and SAA_1_ concentrations in men who developed anaemia, as well as non-anaemia and anaemia men who were tested with CBC three and more times.

We also conducted correlation analyses and discovered a negative, statistically significant relationship between TNF-α concentration and haemoglobin concentration (r = −0.51, *p* = 0.016), haematocrit value (r = −0.53, *p* = 0.011), and red blood cell count (r = −0.61, *p* = 0.002) from the first CBC examination, as well as between TNF-α concentration and the number of red blood cells (r = −0.44, *p* = 0.040) in the third CBC examination. There was also a negative, statistically significant relationship between CRP and haemoglobin concentrations (r = −0.51, *p* = 0.021) from the second CBC examination. Additionally, we found a positive, statistically significant relationship between hepcidin concentration and haemoglobin concentration (r = 0.46, *p* = 0.033), haematocrit value (r = 0.44, *p* = 0.040), and the number of red blood cells (r = 0.43, *p* = 0.044). All correlations are listed in Table 3.

### 3.6. Diagnostic Value of Hepcidin, IL-6, TNF-α, and CRP

We supplemented our findings with a statistical analysis of the diagnostic value of hepcidin, IL-6, TNF-a, CRP, and SAA_1_ in the examination and differentiation of anaemia in lung cancer patients. Among the examined models, we discovered a statistically significant diagnostic model consisting of hepcidin and IL-6 in the differential diagnosis of females without anaemia from females who will develop anaemia post-operatively during the hospitalization, with the highest sensitivity (87.5%) and specificity (71.4%), as well as an area under the curve (AUC) value considered excellent (0.8809).

**Figure 7:**
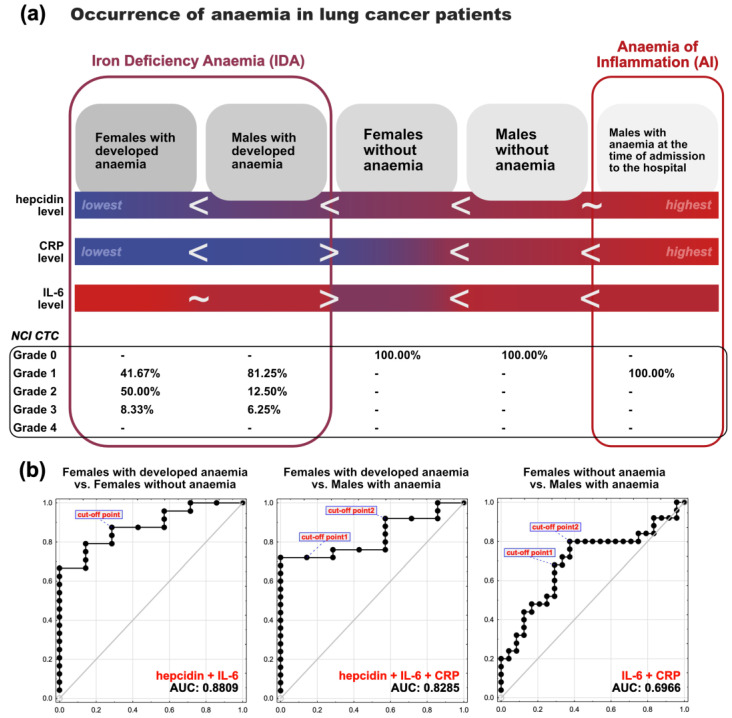
The potential diagnostic model for (**a**) determining the foundation in the pathogenesis of anaemia in lung cancer patients, as well as predicting anaemia severity, and (**b**) distinguishing patients with anaemia since hospital admission from patients with anaemia developed during hospitalization from patients without anaemia during hospitalization.

We also found statistically significant diagnostic models when distinguishing (1) males without anaemia from males with anaemia since hospital admission, (2) females who will develop anaemia post-operatively from males with anaemia since hospital admission, and (3) females without anaemia from males with anaemia since hospital admission. Table 7 shows the diagnostic efficiency of the best biomarker models for differential diagnosis in each and every group of lung cancer patients studied, whereas Figure 7b shows receiver operating characteristic (ROC) curves of the three statistically significant models with the highest sensitivity, specificity, and AUC values.

## 4. Discussion

In clinical practice, anaemia is not a problem in cancer patients until it is severe or life-threatening anaemia, at which point therapeutic actions are taken. Researchers are also focusing on severe or life-threatening anaemia, particularly in terms of the side effects of used chemotherapy or radiotherapy [8,19,20,21]. To expose the lack of interest/negligibility of the anaemia problem in lung cancer patients, we obtained the following numbers after searching the PubMed database. After conducting a keyword search on 23 September 2022: (1) (lung cancer OR lung neoplasms OR lung tumour) AND (anaemia OR anemia), we found 3547 results; (2) (lung cancer OR lung neoplasms OR lung tumour) AND (anaemia OR anemia) AND chemotherapy, we found 2282 results; (3) (lung cancer OR lung neoplasms OR lung tumour) AND (anaemia OR anemia) AND radiotherapy, we found 316 results; (4) (lung cancer OR lung neoplasms OR lung tumour) AND hepcidin, we found 22 results, compared to 432,949 results after searching for lung cancer OR lung tumour OR lung neoplasm.

Lung cancer patients not only have the highest incidence of anaemia, but they also have the highest mortality rate, with low overall five-year relative survival rates [2,5,13]. Given that anaemia is independently associated with shorter cancer patient survival times, even mild or moderate anaemia should be a significant issue in lung cancer patients [9,12,13].

In the present study, we focused on anaemia associated with the condition of lung cancer, as well as anaemia that developed in lung cancer patients post-operatively. We discovered that males outnumbered females in the group of patients admitted to the hospital with anaemia. Anaemia was found in 29.46% of 112 cancer patients at the time of admission to the hospital, including 45.07% of 71 male cancer patients and 2.44% of 41 female cancer patients. We also found that the anaemia group had a higher mean age. All of these patients with anaemia since hospital admission had mild (grade 1) anaemia, according to the NCI grading. Furthermore, 35.44% of the 79 remaining lung cancer patients who did not have anaemia at the time of admission to the hospital developed anaemia in the course of their hospitalization, including 41.03% of 39 male cancer patients and 30.00% of 40 female cancer patients. In contrast to the group with anaemia since hospital admission, we observed a variety of anaemia severity in the “developed anaemia group”, with patients having mild, moderate, or severe anaemia. We found that females with developed anaemia had a higher proportion of moderate and severe anaemia than males with developed anaemia.

Statistical analyses revealed that males with anaemia on admission to the hospital were not only older than non-anaemia patients in our study group, but they also had the highest CRP concentrations, above reference values (>5 mg/L). The current state of the art indicates that comparing hepcidin to CRP may be a quick and simple way to determine whether anaemia is the result of an iron deficiency, an inflammation, or a mixture of both [15,22]. Hence, increased hepcidin concentrations in combination with high CRP concentrations enable us to determine the presence of inflammation in the pathogenesis of anaemia in lung cancer males admitted to the hospital with anaemia.

Anaemia of inflammation is also known as anaemia of chronic disease because it occurs in people who have chronic conditions that may be associated with inflammation. Cancer is one of these chronic conditions. [15] Anaemia of inflammation is more common in the elderly and may be associated with the term inflammageing coined by Claudio Franceschi in ~2000 [16,17,23]. Our finding confirmed the concept of inflammaeging and anaemia of inflammation, as people with anaemia on hospital admission were on average 5 years older than non-anaemia patients.

Franceschi and colleagues [23] reported an elevated basal level of pro-inflammatory mediators in the blood even in healthy elderly people. High circulating levels of IL-1, IL-1 receptor antagonist protein (IL-1RN), IL-6, IL-8, IL-13, IL-18, CRP, IFN-α, IFN-β, TGF-β, TNF-α and its soluble receptors, and SAA_1_ characterise this mild pro-inflammatory state. Furthermore, cancer itself also stimulates the production of pro-inflammatory cytokines. [12,14,16,17,24,25] TNF-α and IL-6 cytokines are secreted by non-small cell lung cancer (NSCLC) cells and immune cells infiltrating the tumour microenvironment, and they regulate liver cell production of the non-specific acute phase proteins CRP and SAA_1_ [26]. In addition, IL-6 regulates hepcidin expression through the direct binding of STAT3 (signal transducer and activator of transcription 3) to the promoter. As a result, chronic IL-6 elevation is involved in the development of anaemia, among other things, by increasing hepcidin production and thus serum hepcidin levels. Both cytokines and hepcidin have been shown to inhibit erythropoiesis by (1) storing iron in reticuloendothelial system cells, (2) decreasing iron absorption in the digestive system, (3) inhibiting erythropoietin production in the kidneys, and (4) directly inhibiting proliferation and differentiation of erythroid progenitor cells [10,12,15,22,25,27].

Furthermore, other studies show that healthy women have higher IL-6 serum levels later in life than healthy men, implying that women experience the effects of inflammageing later than men [23,28]. Because lung cancer males have additive effects on pro-inflammatory cytokine levels from inflammageing and cancer, and lung cancer females have higher concentrations of pro-inflammatory cytokines from cancer itself, we may conclude that anaemia of inflammation is more common in older male lung cancer patients. This conclusion would explain why men outnumber women in the group of patients with anaemia upon admission to the hospital (45.07% of all males vs. 2.44% of all females).

We also observed that patients who developed anaemia post-operatively were characterized by significantly lower hepcidin concentrations than non-anaemic patients and patients who had anaemia since their hospital admission. A low hepcidin concentration combined with a low CRP concentration (within reference values, <5 mg/L) indicates the presence of iron deficiency in the pathogenesis of anaemia [15], in this case, anaemia that develops during the patient’s hospitalization. Moreover, we found that female patients who developed anaemia during hospitalization had a higher frequency of moderate or severe anaemia than male patients. Additionally, females who developed anaemia had statistically lower hepcidin concentrations than males. We concluded that the severity of developed anaemia, which is characterized by iron deficiency in its pathogenesis, is related to the patient’s hepcidin level, i.e., lower hepcidin concentrations are associated with more severe anaemia.

While hepcidin and CRP examination may point to the pathogenesis of anaemia, we discovered that hepcidin and IL-6 examination may predict the development of anaemia during hospitalization in female lung cancer patients. The model with (area under the curve) AUC = 0.8809 provides good discrimination and allows differentiation of women who will not develop anaemia from women who will develop anaemia during the hospitalization, with a high sensitivity and specificity. Based on our observations, section (a) of Figure 7 depicts a scheme of a possible diagnostic procedure in differentiation and determination of the foundation in the pathogenesis of anaemia in lung cancer patients, as well as the prediction of anaemia severity. While section (b) of Figure 7 depicts receiver operating characteristic (ROC) curves assessing the ability of multi-biomarker models to distinguish (1) women who will not develop anaemia from women who will develop anaemia during the hospitalization, (2) men with anaemia from women who will develop anaemia during the hospitalization, and (3) men with anaemia from women who will not develop anaemia during the hospitalization.

An algorithm for diagnosing and predicting anaemia in lung cancer patients is required. The ECAS was one of the first to raise awareness of the significance of mild-to-moderate anaemia on patients who may not be treated according to Anaemia Society of Haematology/American Society of Clinical Oncology (ASH/ASCO) guidelines. Even mild-to-moderate anaemia, according to ECAS, has a significant impact on a patient’s QoL and performance status, as measured by the physician-reported WHO Performance Score [9]. Anaemia in cancer patients causes micro changes such as tumour hypoxia and the production of angiogenic factors, both of which control further tumour growth and progression, and have been linked to the development of treatment resistance [12,20]. In addition to resistance to chemo- and radiotherapy, anaemia of cancer is responsible for increased post-operative mortality, impaired organ function, decreased QoL, and poor prognoses [11,13]. Cancer patients with haemoglobin levels less than 12 g/dL have significantly more fatigue, more non-fatigue anaemia symptoms, poorer physical, functional, and mental well-being, and lower general QoL [12]. Furthermore, a Southwest Oncology Group study that enrolled patients with advanced-stage NSCLC between 1974 and 1988 discovered that patients with haemoglobin levels greater than 11 g/dL had a better prognosis [13,29]. There is also evidence that higher haemoglobin levels, even when increased by epoetin, are associated with better outcome measures such as survival [12,13].

When mild or moderate anaemia appears, there is a lack of awareness about the potential consequences for patients’ quality of life and overall survival. Clinicians must become more aware of the clinical impact of not only severe and life-threatening anaemia, but also mild and moderate anaemia, and offer adequate treatment to their patients [11]. At this point, anaemia treatment should be a critical component of cancer treatment. Especially since we can predict which operative lung cancer patients will develop anaemia during hospitalization using hepcidin, IL-6, and CRP testing and include these patients in anaemia prevention treatment. In the short term, preventing and treating anaemia in lung cancer patients may improve post-operative recovery as well as shorten the duration of the patient’s hospitalization, as patients with developed anaemia spent 2 to 4 days longer in the hospital on average. In the long term, preventing and treating anaemia in lung cancer patients may be another key point in improving their survival rates, resulting in decreased lung cancer patient mortality. Overall, prevention and treatment of anaemia in lung cancer patients appear to be beneficial for the patient themself and from the economical point of view.

Our main findings demonstrate and confirm the role of hepcidin and IL-6 in the pathogenesis of lung cancer-related anaemia, as well as present diagnostic values for hepcidin, IL-6, and CRP. TNF-α, however, cannot be ruled out as a factor in the anaemia of cancer. Numerous studies described the central role of TNF-α in the pathogenesis of anaemia [10,12,24]. TNF-α’s inhibitory effect on erythroid differentiation was first described over 30 years ago. Blick and colleagues [30] discovered in 1987 a decrease in haemoglobin synthesis in cancer patients treated with TNF-α, and an in vitro study revealed that TNF-α inhibited the formation of burst-forming unit-erythrocyte (BFU-E) cells.

In the present study, we discovered statistical significance in the model consisting of TNF-α alone in distinguishing men without anaemia during hospitalization from men with anaemia since hospital admission, with men with anaemia since hospital admission, recognized by us as anaemia of inflammation, having higher TNF-α concentrations. Correlation analysis confirmed these findings. TNF-α concentration was found to be negatively correlated with CBC parameters examined upon hospital admission: haemoglobin concentration, haematocrit value, and red blood cell count, implying that the values of CBC parameters were decreased in patients with higher TNF-α concentration. The significance of TNF-α in our study group’s males, with a focus on males with anaemia since hospital admission, may point to TNF-α’s role in the pathogenesis of anaemia of inflammation. The activation of STAT3 by IL-6 regulates hepcidin expression. However, because STAT3 can be activated by a variety of other cytokines and growth factors, hepcidin expression may be influenced by a variety of other factors, including TNF-α [22,25,27].

Serum hepcidin concentrations and local hepcidin expression have been reported to increase in a variety of neoplasms, including lung cancer, breast cancer, renal carcinoma, prostate cancer, colorectal cancer, and acute leukaemia. Elevated serum hepcidin levels and local expression have been linked to disease progression, cancer metastatic potential, and shorter overall survival in oncological patients [31,32,33]. Despite knowledge and awareness of the role and potential consequences of increased hepcidin levels in oncological patients, there is a lack of studies focusing on both the potential diagnostic or therapeutic use of hepcidin and its role in the development of cancer-related anaemia, as well as how to incorporate this ready-to-use marker into the clinical field.

Our study is the first to investigate hepcidin’s diagnostic capabilities in lung cancer anaemia in a comprehensive and detailed manner. We discovered that hepcidin reacts differently in lung cancer patients with anaemia. We linked two hepcidin roles with two cancer anaemia pathogeneses. We found higher hepcidin concentrations in patients with anaemia since hospital admission, which we labelled as anaemia of inflammation in its pathogenesis, not related to lung cancer treatment (in our case, surgery), but to the cancer itself. On the other hand, we found lower levels of hepcidin in patients who developed anaemia after surgery, which we labelled anaemia of iron deficiency in its pathogenesis. We proposed a diagnostic strategy that would enable clinicians to predict which lung cancer patients are at risk of developing anaemia after surgery. This type of strategy would aid in the reduction of post-operative side effects while also increasing the overall survival of lung cancer patients.

Our study had a few limitations, including the lack of iron distribution analysis in our lung cancer patients. We also struggled with a scarcity of comparable studies that could confirm or refute our hypotheses and findings. Despite meeting the high power of the power analysis test in the key examinations that supported our hypotheses, some analyses had insufficient power analysis. The number of patients in our study group should be increased for this purpose. Moreover, not every patient had a clear and defined endpoint. We reduced the risk of incorrectly classifying patients who might have developed anaemia during their hospitalization as non-anaemia patients but having a full study group with a minimum of three CBCs examinations: upon admission to the hospital, on the day of surgery after surgery, and 3–4 days later would be the perfectly designed study for our analyses. Given these comments, our study should be regarded as a pilot study.

## 5. Conclusions

There is a need to incorporate hepcidin as a diagnostic marker into clinical practice, which necessitates the development of a quantitative, sensitive, quick, easy, and low-cost diagnostic method for routine diagnosis in diagnostic laboratories. In future studies, the cut-off values, at which hepcidin, IL-6, and CRP concentrations may differentiate future anaemia patients from patients who are not expected to develop anaemia, must be determined.

## Figures and Tables

**Table 1:** Definition of anaemia and its severity according to WHO/NCI classification/grading.

Severity of Anaemia	NCI CTC/WHO Grading	Haemoglobin Levels [g/dL]	
		NCI References	WHO References
No anaemia	0	Normal	≥11
Mild	1	10 to upper limit of normal	9.5–10.9
Moderate	2	8.0–9.9	8.0–9.4
Severe	3	6.5–7.9	6.5–7.9
Life-threatening	4	<6.5	<6.5

NCI CTC—National Cancer Institute Common Toxicity Criteria; WHO—World Health Organisation.

**Table 2:** Lung cancer patients’ characteristics divided by gender.

	Women	Men	Men with Anaemia upon Admission to the Hospital	Overall
N (%)	41 (36.61%)	71 (63.39%)	32 (28.57%)	112 (100%)
Age				
Mean ± SD	66.2 ± 8.3	67.8 ± 8.2	71.1 ± 6.5	67.2 ± 8.2
Range	40–81	39–84	58–84	39–84
Median	67	69	73	68
Surgery [n, (%)]				
Lobectomy	30 (73.17%)	31 (43.66%)	16 (50.00%)	61 (54.46%)
Wedge Resection	8 (19.51%)	19 (26.76%)	9 (28.13%)	27 (24.11%)
Biopsy	-	10 (14.08%)	5 (15.63%)	10 (8.93%)
Segmentectomy	2 (4.88%)	5 (7.04%)	1 (3.13%)	7 (6.25%)
Bilobectomy	1 (2.44%)	4 (5.63%)	1 (3.13%)	5 (4.46%)
Pulmonectomy	-	2 (2.82%)	-	2 (1.79%)
Lung cancer subtype [n, (%)]				
Adenocarcinoma	21 (51.22%)	29 (40.85%)	13 (40.63%)	50 (44.64%)
Squamous cell carcinoma	8 (19.51%)	27 (38.03%)	12 (37.50%)	35 (31.25%)
Other NSCLC	5 (12.20%)	8 (11.27%)	4 (12.50%)	13 (11.61%)
Not NSCLC	7 (17.07%)	7 (9.86%)	3 (9.38%)	14 (12.50%)
Stage [n, (%)]				
IA	14 (34.15%)	11 (15.49%)	4 (12.50%)	25 (22.32%)
IB	9 (21.95%)	9 (12.68%)	5 (15.63%)	18 (16.07%)
IIA	-	5 (7.04%)	1 (3.13)	5 (4.46%)
IIB	9 (21.95%)	12 (16.90%)	7 (21.88%)	21 (18.75%)
IIIA	2 (4.88%)	10 (14.08%)	4 (12.50%)	12 (10.71%)
IIIB	1 (2.44%)	10 (14.08%)	5 (15.63%)	11 (9.82%)
IVA	4 (9.76%)	11 (15.49%)	4 (12.50%)	15 (13.39%)
IVB	-	-	-	-
Grading [n, (%)]				
G1	1 (2.44%)	-	-	1 (0.89%)
G2	17 (41.46%)	28 (39.44%)	13 (40.63%)	45 (40.18%)
G3	8 (19.51%)	12 (16.90%)	5 (15.63%)	20 (17.86%)
NA	15 (36.59%)	31 (43.66%)	14 (43.75%)	46 (41.07%)
Inflammation in the tumour environment [n, (%)]				
YES	19 (46.34%)	28 (39.44%)	13 (40.63%)	47 (41.96%)
NO	22 (53.66%)	43 (60.56%)	19 (59.38%)	65 (58.04%)
Smoking history [n, (%)]				
Current	11 (26.83%)	20 (28.17%)	7 (21.88%)	31 (27.68%)
Former	18 (43.90%)	36 (50.70%)	18 (56.25%)	54 (48.21%)
Passive	2 (4.88%)	-	-	2 (1.79%)
Never	1 (2.44%)	1 (1.41%)	1 (3.13%)	2 (1.79%)
NA	9 (21.95%)	14 (19.72%)	6 (18.75%)	23 (20.54%)
Comorbidities [n, (%)]				
YES	26 (63.41%)	47 (66.20%)	22 (68.75%)	73 (65.18%)
NO	15 (36.59%)	24 (33.80%)	10 (31.25%)	39 (34.82%)
Type of comorbidity [n, (%)]				
NO	15 (36.59%)	24 (33.80%)	10 (31.25%)	39 (34.82%)
DM	1 (2.44%)	2 (2.82%)	1 (3.13%)	3 (2.68%)
COPD	2 (4.88%)	4 (5.63%)	2 (6.25%)	6 (5.36%)
Hypertension	16 (39.02%)	22 (30.99%)	9 (28.13%)	38 (33.93%)
DM + Hypertension	5 (12.20%)	9 (12.68%)	5 (15.63%)	14 (12.50%)
COPD + Hypertension	1 (2.44%)	7 (9.86%)	3 (9.38%)	8 (7.14%)
DM + COPD + Hypertension	1 (2.44%)	3 (4.23%)	2 (6.25%)	4 (3.57%)

SD—standard deviation; IA-IVB—stages of lung cancer based on the AJCC 8th TNM Staging System; NA—not available; G1—grade 1, well-differentiated; G2—grade 2, moderately differentiated; G3—grade 3, poorly differentiated; DM—diabetes mellitus; COPD—chronic obstructive pulmonary disease.

**Table 3:** List of statistically important correlations between analysed parameters in women and men with one complete blood count examination, as well as ≥3 complete blood count examinations.

	Hepcidin [ng/mL]	IL-6 [pg/mL]	TNF-α [pg/mL]	CRP [mg/L]	SAA_1_ [μg/mL]
Patients with one complete blood count examination					
Women					
Hepcidin		r = 0.99, *p* = 0.045			
IL-6	r = 0.99, *p* = 0.045		r = 0.99, *p* = 0.026		
TNF-α		r = 0.99, *p* = 0.026			
Men					
Hb from 1st CBC				r = −0.96, *p* = 0.035	
RBC from 1st CBC			r = −0.99, *p* = 0.014		
Patients with two complete blood count examinations					
Women					
TNF-α					r = 0.55, *p* = 0.013
SAA_1_			r = 0.55, *p* = 0.013		
Hospital stay				r = 0.57, *p* = 0.008	
Men					
CRP					r = 0.51, *p* = 0.018
SAA_1_				r = 0.51, *p* = 0.018	
Patients with ≥3 complete blood count examinations					
Women					
CRP					r = 0.92, *p* = 0.000
SAA_1_				r = 0.92, *p* = 0.000	
RBC from 1st CBC	r = −0.75, *p* = 0.019				
Hb from 3rd CBC	r = 0.73, *p* = 0.026				
Ht from 3rd CBC	r = 0.72, *p* = 0.029				
RBC from 3rd CBC	r = 0.70, *p* = 0.036				
Men					
Hb from 1st CBC			r = −0.51, *p* = 0.016		
Ht from 1st CBC			r = −0.53, *p* = 0.011		
RBC from 1st CBC			r = −0.61, *p* = 0.002		
Hb from 2nd CBC				r = −0.51, *p* = 0.021	
Hb from 3rd CBC	r = 0.46, *p* = 0.033				
Ht from 3rd CBC	r = 0.44, *p* = 0.040				
RBC from 3rd CBC	r = 0.43, *p* = 0.044		r = −0.44, *p* = 0.040		

IL-6—interleukin 6; TNF-α—tumour necrosis factor α; CRP—C-reactive protein; SAA_1_—serum amyloid A_1_; CBC—complete blood count; RBC—red blood count; Hb—haemoglobin; Ht—haematocrit; r—correlation coefficient measuring the degree of correlation.

**Table 4:** Descriptive statistics of complete blood count parameters as well as hepcidin, IL-6, TNF-α, CRP, and SAA_1_ levels in women with and without post-operative anaemia in the third complete blood count examination.

	The Prevalence of Anaemia 3–4 Days after Surgery			
	No Anaemia (N = 4)		Developed Anaemia (N = 10)	
Parameter	1st CBC	3rd CBC	1st CBC	3rd CBC
NCI grading [n (%)]				
Grade 0	4 (100%)	4 (100%)	10 (100%)	-
Grade 1	-	-	-	3 (30%)
Grade 2	-	-	-	6 (60%)
Grade 3	-	-	-	1 (10%) *
Grade 4	-	-	-	-
Hb [g/dL]				
Mean ± SEM	12.63 ± 0.43	12.28 ± 0.36 ^a^	12.64 ± 0.21	9.27 ± 0.35 ^a^
Median	12.55	12.3	12.5	9.3
Min–Max	11.70–13.70	11.60–12.90	11.30–13.50	7.20–10.70
RBC [mln/μL]				
Mean ± SEM	4.09 ± 0.08	4.02 ± 0.08 ^b^	4.31 ± 0.09	3.18 ± 0.13 ^b^
Median	4.11	3.98	4.25	3.24
Min–Max	3.89–4.24	3.89–4.24	3.94–4.72	2.51–3.82
Hepcidin [ng/mL]				
Mean ± SEM	331.43 ± 54.41 ^c^		99.34 ± 17.56 ^c^	
Median	351.96		116.35	
Min–Max	181.01–440.77		16.18–143.47	
IL-6 [pg/mL]				
Mean ± SEM	24.64 ± 4.72 ^d^		53.72 ± 7.68 ^d^	
Median	21.15		50.36	
Min–Max	17.81–38.45		34.37–99.69	
TNF-α [pg/mL]				
Mean ± SEM	4.22 ± 2.87		3.17 ± 0.65	
Median	1.54		2.84	
Min–Max	0.99–12.84		1.85–6.87	
CRP [mg/L]				
Mean ± SEM	33.86 ± 15.89		4.76 ± 1.64	
Median	29.74 ^e^		1.85 ^e^	
Min–Max	1.96–74.01		0.73–16.42	
SAA_1_ [μg/mL]				
Mean ± SEM	256.28 ± 155.64		68.03 ± 30.14	
Median	194.47		18.64	
Min–Max	0.32–635.87		0.50–282.63	

CBC—complete blood count; NCI—National Cancer Institute; Hb—haemoglobin; RBC—red blood count; IL-6—interleukin 6; TNF-α—tumour necrosis factor α; CRP—C-reactive protein; SAA_1_—serum amyloid A_1_; SEM—standard error of the mean. Statistical significances, Student’s *t*-test: ^a^ *p* = 0.000312 in 3rd CBC examination haemoglobin concentrations between women with and without anaemia after surgery; ^b^ *p* = 0.000217 in 3rd CBC examination RBC values between women with and without anaemia after surgery; ^c^ *p* = 0.000694 between women with and without anaemia after surgery in hepcidin concentrations; ^d^ *p* = 0.030727 between women with and without anaemia after surgery in IL-6 concentrations; Mann–Whitney U test: ^e^ *p* = 0.040306 between women with and without anaemia after surgery in CRP concentrations; chi-square test: * *p* = 0.030522 between females and males who developed anaemia during their hospitalization, in the frequency of developing grade 1 and 2 anaemia.

**Table 5:** Descriptive statistics of age and hepcidin, IL-6, TNF-α, CRP, and SAA_1_ levels in lung cancer patients, subdivided by the presence of anaemia at the time of admission to the hospital.

	Anaemia upon Admission to the Hospital		
Parameter	Non-Anaemia Women	Non-Anaemia Men	Anaemia Men
Age (years)			
Mean ± SD	66.1 ± 8.3 ^a^	66.1 ± 7.5 ^b^	71.1 ± 6.5 ^a,b^
Median	66.5	67	73
Min–Max	40.0–81.0	47.0–82.0	58.0–84.0
Hepcidin (ng/mL)			
Mean ± SEM	125.27 ± 16.78 ^c^	174.64 ± 16.86 ^c^	169.02 ± 24.05
Median	109.16	161.5	147.36
Min–Max	11.94–440.77	31.44–429.52	0.08–525.15
IL-6 (pg/mL)			
Mean ± SEM	30.85 ± 3.46	33.49 ± 4.99	36.23 ± 4.50
Median	27.74	26.78	34.95
Min–Max	0.32–99.69	0.21–113.83	0.36–112.70
TNF-α (pg/mL)			
Mean ± SEM	2.64 ± 0.36	6.15 ± 2.96	3.18 ± 0.66
Median	2.1	2.18	2.29
Min–Max	0.99–12.84	1.29–111.55	0.57–20.57
CRP (mg/L)			
Mean ± SEM	10.06 ± 2.95	24.40 ± 5.12	41.53 ± 9.60
Median	2.27 ^d,e^	7.56 ^d^	15.06 ^e^
Min–Max	0.25–84.72	0.22–106.44	0.60–217.67
SAA_1_ (μg/mL)			
Mean ± SEM	101.30 ± 22.26	107.99 ± 23.88	132.95 ± 26.39
Median	45.63	7.58	110
Min–Max	0.21–635.87	0.03–548.31	0.63–636.77

SD—standard deviation; SEM—standard error of the mean; IL-6—interleukin 6; TNF-α—tumour necrosis factor α; CRP—C-reactive protein; SAA_1_—serum amyloid A_1_. Statistical significances, One-way ANOVA: ^a^ *p* = 0.007810 between non-anaemia women and anaemia men in patients’ age; ^b^ *p* = 0.004810 between non-anaemia men and anaemia men in patients’ age; ^c^ *p* = 0.041324 between non-anaemia women and non-anaemia men in hepcidin concentrations. One-way ANOVA on ranks (Kruskal–Wallis test by ranks): ^d^ *p* = 0.044504 between non-anaemia women and non-anaemia men in CRP concentrations; ^e^ *p* = 0.002675 between non-anaemia women and anaemia men in CRP concentrations.

**Table 6:** Descriptive statistics of complete blood count parameters from the first and third complete blood count examinations, as well as hepcidin, IL-6, TNF-α, CRP, and SAA_1_ levels in men with and without anaemia, and in men who developed anaemia after surgery.

	The Prevalence of Anaemia 3–4 Days after Surgery					
	No Anaemia (N = 3)		Developed Anaemia (N = 9)		Anaemia (N = 14)	
Parameter	1st CBC	3rd CBC	1st CBC	3rd CBC	1st CBC	3rd CBC
NCI grading (n (%))						
Grade 0	3 (100.0%)	3 (100.0%)	9 (100.0%)	-	-	-
Grade 1	-	-	-	6 (66.7%)	14 (100.0%)	10 (71.4%)
Grade 2	-	-	-	2 (22.2%)	-	4 (28.6%)
Grade 3	-	-	-	1 (11.1%) *	-	-
Grade 4	-	-	-	-	-	-
Hb (g/dL)						
Mean ± SEM	15.37 ± 0.45 ^a,b^	13.80 ± 0.12 ^d,e^	14.30 ± 0.24 ^a,c^	10.72 ± 0.62 ^d^	12.27 ± 0.25 ^b,c^	10.56 ± 0.32 ^e^
Median	15.6	13.8	14	10.7	12.55	11.1
Min–Max	14.50–16.00	13.60–14.00	13.70–15.90	7.60–13.30	10.20–13.30	8.50–12.00
RBC (mln/μL)						
Mean ± SEM	4.94 ± 0.17 ^f^	4.41 ± 0.07 ^h,i^	4.89 ± 0.10 ^g^	3.71 ± 0.27 ^h^	4.06 ± 0.08 ^f,g^	3.52 ± 0.10 ^i^
Median	5.03	4.39	4.79	3.75	4.07	3.53
Min–Max	4.61–5.19	4.31–4.54	4.55–5.33	2.69–5.04	3.51–4.53	2.92–3.99
Hepcidin (ng/mL)						
Mean ± SEM	251.19 ± 58.30		240.74 ± 41.34		214.84 ± 34.74	
Median	247.96		259.62		188.37	
Min–Max	151.86–353.74		78.61–429.52		60.85–525.15	
IL-6 (pg/mL)						
Mean ± SEM	67.45 ± 18.45		52.93 ± 12.34		45.82 ± 6.95	
Median	64.62		46.14		45.32	
Min–Max	37.00–100.72		0.21–113.83		0.36–112.70	
TNF-α (pg/mL)						
Mean ± SEM	1.99 ± 0.27		1.98 ± 0.15		2.71 ± 0.30	
Median	2.2		1.91		2.54	
Min–Max	1.45–2.33		1.44–2.71		1.20–4.53	
CRP (mg/L)						
Mean ± SEM	54.24 ± 21.44		29.49 ± 11.10		48.05 ± 16.90	
Median	65.52		17.33		13.7	
Min–Max	12.78–84.43		1.44–81.32		0.81–217.67	
SAA_1_ (μg/mL)						
Mean ± SEM	59.44 ± 55.68		162.43 ± 61.76		146.22 ± 50.08	
Median	7.58		101.35		57.12	
Min–Max	0.03–170.72		0.08–515.75		0.63–636.77	

CBC—complete blood count; NCI—National Cancer Institute; Hb—haemoglobin; RBC—red blood count; IL-6—interleukin 6; TNF-α—tumour necrosis factor α; CRP—C-reactive protein; SAA_1_—serum amyloid A_1_; SEM—standard error of the mean. Statistical significances, one-way ANOVA: ^a^ *p* = 0.045056 in haemoglobin concentration upon hospital admission between men who did not have anaemia and men who developed anaemia post-operatively; ^b^ *p* = 0.000138 in haemoglobin concentration upon hospital admission between men who did not have anaemia and men who had anaemia; ^c^ *p* = 0.000653 in haemoglobin concentration upon hospital admission between men who developed anaemia post-operatively and men who had anaemia; ^d^ *p* = 0.002236 in haemoglobin concentration from the 3rd CBC examination between men who did not have anaemia and men who developed anaemia post-operatively; ^e^ *p* = 0.003834 in haemoglobin concentration from the 3rd CBC examination between men who did not have anaemia and men who had anaemia; ^f^ *p* = 0.000199 in RBC values upon hospital admission between men who did not have anaemia and men who had anaemia; ^g^ *p* = 0.000212 in RBC values upon hospital admission between men who developed anaemia post-operatively and men who had anaemia; ^h^ *p* = 0.040572 in RBC values from the 3rd CBC examination between men who did not have anaemia and men who developed anaemia post-operatively; ^i^ *p* = 0.028428 in RBC values from the 3rd CBC examination between men who did not have anaemia and men who had anaemia; chi-square test: * *p* = 0.030522 between females and males who developed anaemia during their hospitalization, in the frequency of developing grade 1 and 2 anaemia.

**Table 7:** The diagnostic efficiency of biomarker models to differentiate lung cancer patients without anaemia, patients with anaemia since the day of hospitalization, and patients who developed anaemia during their hospitalization based on patient gender.

Model	*p* Value	AIC	AUC	Sensitivity	Specificity	Cut-Off Values
Females without anaemia vs. Females with developed anaemia						
Hepcidin + IL-6	0.00574	28.8	0.8809	87.50%	71.40%	Hepcidin: 44.29 ng/mL IL-6: 24.39 pg/mL
Males without anaemia vs. Males with developed anaemia						
IL-6	0.171698	40.37	0.6458	75.00%	50.00%	IL-6: 18.77 pg/mL
Males without anaemia vs. Males with anaemia						
TNF-α	0.032369	54.27	0.6687	72.00%	56.30%	TNF-α: 2.59 pg/mL
Males with anaemia vs. Males with developed anaemia						
IL-6	0.448175	50.05	0.5533	60.00%	50.00%	IL-6: 39.58 pg/mL
Females with developed anaemia vs. Males with anaemia						
Hepcidin + IL-6 + CRP	0.00927	30.11	0.8285	(1) 72.0%	(1) 85.7%	(1) Hepcidin: 66.16 ng/mL
						IL-6: 57.61 pg/mL
						CRP: 21.23 mg/L
				(2) 92.0%	(2) 42.9%	(2) Hepcidin: 0.08 ng/mL
						IL-6: 2.63 pg/mL
						CRP: 0.60 mg/L
Females without anaemia vs. Males with anaemia						
IL-6 + CRP	0.02763	66.73	0.6966	(1) 68.0%	(1) 70.8%	(1) IL-6: 44.14 pg/mL
						CRP: 0.88 mg/L
				(2) 80.0%	(2) 62.5%	(2) IL-6: 34.95 pg/mL
						CRP: 0.60 mg/L

AIC—Akaike information criterion; AUC—area under the curve; IL-6—interleukin 6; TNF-α—tumour necrosis factor α; CRP—C-reactive protein; bold black text indicates statistically significant diagnostic model.

## Data Availability

The data presented in this study are available on request from the corresponding author. The data are not publicly available due to privacy and ethical restriction.

## Supplementary Materials

The following supporting information can be downloaded at: https://www.mdpi.com/article/10.3390/cancers15010224/s1, Figure S1: The prevalence of anaemia in women with operable lung cancer before and after surgery; Figure S2: The prevalence of anaemia in men with operable lung cancer before and after surgery; Table S1: Complete blood count parameters determined during hospitalization of women and men with operable lung cancer (dynamics of changes); Table S2: Hepcidin, IL-6, TNF-α, CRP, and SAA_1_ concentrations in non-anaemia and anaemia women who had one complete blood count examination (upon hospital admission); Table S3: Hepcidin, IL-6, TNF-α, CRP, and SAA_1_ concentrations in non-anaemia women and women with post-operative anaemia who had two complete blood counts; Table S4: Hepcidin, IL-6, and TNF-α concentrations in (a) males with anaemia since hospital admission without and with comorbidities, (b) males who developed anaemia during hospitalization without and with comorbidities, and (c) males with no anaemia during hospitalization without and with comorbidities; Table S5: Hepcidin, IL-6, TNF-α, CRP, and SAA_1_ concentrations in non-anaemia and anaemia men who had one complete blood count examination (upon hospital admission); Table S6: Descriptive statistics of complete blood count parameters from the first and second complete blood count examinations, as well as hepcidin, IL-6, TNF-α, CRP, and SAA_1_ levels in men with and without anaemia and men with post-operative anaemia.
