# Supplementary material for: Hepcidin as a Diagnostic Biomarker in Anaemic Lung Cancer Patients
Source: Cancers (Basel). 2022 Dec 30;15(1):224. doi: 10.3390/cancers15010224 (PMC9818260; doi:10.3390/cancers15010224)
Supplement: Supplementary file 1 [file cancers-15-00224-s001.zip › cancers-2070970-supplementary.pdf]

Supplementary Materials

Hepcidin as a Diagnostic Biomarker in Anaemic Lung Cancer Patients

Katarzyna Wadowska [1,*], Piotr Błasiak [2,3], Adam Rzechonek [2,3], Iwona Bil-Lula [1] and Mariola Śliwińska-Mossoń [1]

[1] Department of Medical Laboratory Diagnostics, Division of Clinical Chemistry and Laboratory Haematology, Faculty of Pharmacy, Wroclaw Medical University, Borowska 211A, 50-556 Wroclaw, Poland
[2] Department and Clinic of Thoracic Surgery, Faculty of Medicine, Wroclaw Medical University, Grabiszyńska 105, 53-439 Wroclaw, Poland
[3] Lower Silesian Centre of Oncology, Pulmonology and Haematology, Lower Silesian Thoracic Surgery Centre, Grabiszyńska 105, 53-439 Wroclaw, Poland
* Correspondence: katarzyna.wadowska@student.umw.edu.pl; Tel.: +48-71-784-0626

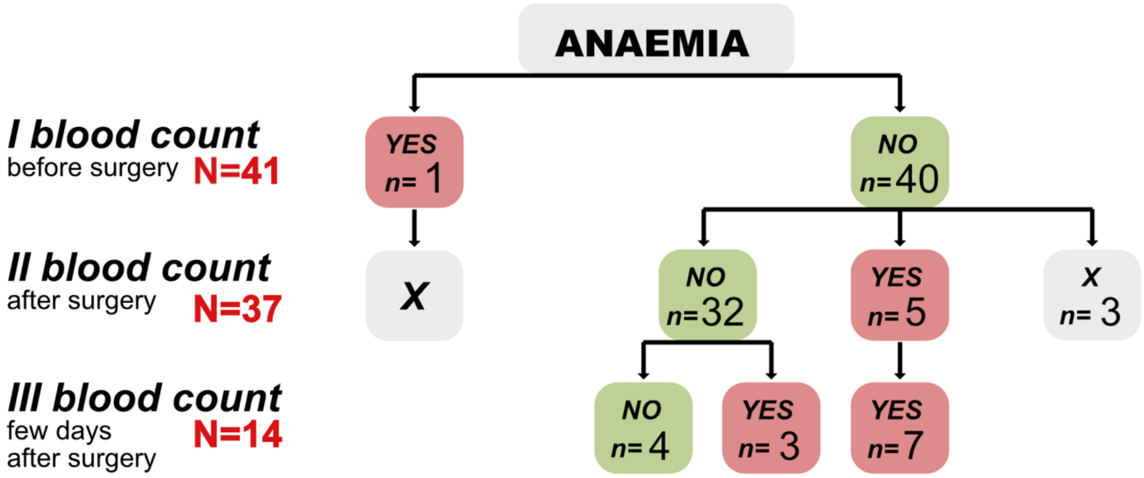

Figure S1. The prevalence of anaemia in women with operable lung cancer before and after surgery.

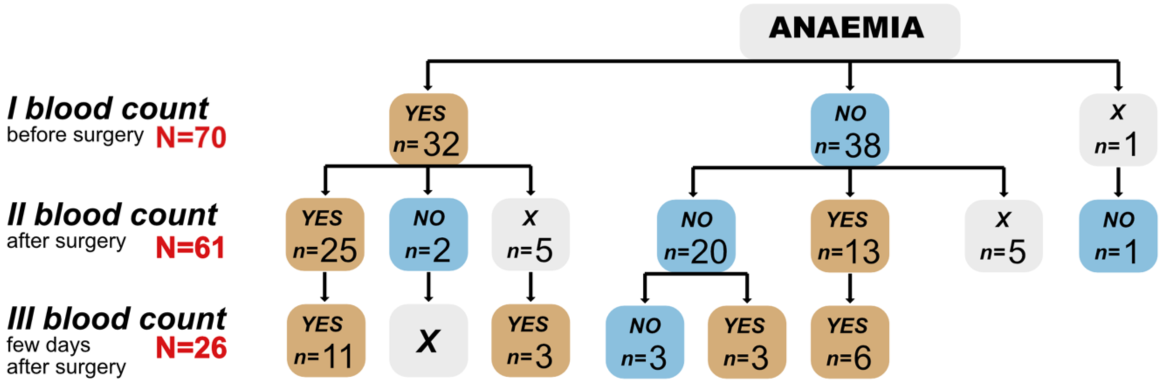

Figure S2. The prevalence of anaemia in men with operable lung cancer before and after surgery.

Table S1. Complete blood count parameters determined during hospitalization of women and men with operable lung cancer (dynamics of changes).

	Before surgery	After surgery (on the day of surgery)	3-4 days after surgery	Laboratory's reference values
**Women**	N=41	N=37	N=14	
Hb [g/dL] ± SEM	13.15 ± 0.20	12.44 ± 0.23	**10.13 ± 0.46**	11.2 – 15.7
Ht [%] ± SEM	39.39 ± 0.51	37.16 ± 0.64	**30.71 ± 1.32**	34 – 45
RBC [10^6/µL] ± SEM	4.37 ± 0.06	4.13 ± 0.08	**3.42 ± 0.14**	3.93 – 5.22
MCV [fL] ± SEM	90.44 ± 0.80	90.16 ± 0.88	90.21 ± 1.33	79 – 95
MCH [pg] ± SEM	30.15 ± 0.31	30.19 ± 0.35	29.64 ± 0.46	26 – 32
MCHC [g/dL] ± SEM	33.33 ± 0.15	33.44 ± 0.17	32.77 ± 0.22	32.2 – 35.5
RDW-SD [fL] ± SEM	44.93 ± 0.66	44.31 ± 0.69	43.46 ± 0.93	36.4 – 46.3
RDW-CV [%] ± SEM	13.96 ± 0.20	13.79 ± 0.21	14.10 ± 0.27	11.7 – 14.4
**Men**	N=71	N=60	N=26	
Hb [g/dL] ± SEM	**13.39 ± 0.18**	**12.71 ± 0.22**	**10.99 ± 0.35**	13.7 – 17.5
Ht [%] ± SEM	**40.19 ± 0.47**	**38.15 ± 0.59**	**33.46 ± 0.99**	40 – 51
RBC [10^6/µL] ± SEM	**4.44 ± 0.06**	**4.20 ± 0.08**	**3.69 ± 0.12**	4.63 – 6.08
MCV [fL] ± SEM	90.90 ± 0.79	91.52 ± 0.82	91.04 ± 0.87	79 – 92
MCH [pg] ± SEM	30.33 ± 0.32	30.51 ± 0.32	29.85 ± 0.35	26 – 32
MCHC [g/dL] ± SEM	33.28 ± 0.15	33.28 ± 0.15	32.75 ± 0.20	32.3 – 36.5
RDW-SD [fL] ± SEM	**46.32 ± 0.52**	**46.16 ± 0.59**	**46.08 ± 0.89**	35.1 – 43.9
RDW-CV [%] ± SEM	14.33 ± 0.16	14.18 ± 0.18	14.31 ± 0.24	11.6 – 14.4

Hb – haemoglobin; Ht – haematocrit; RBC – red blood count; MCV – mean corpuscular volume; MCH – mean corpuscular haemoglobin; MCHC – mean corpuscular haemoglobin concentration; RDW-SD – red cell distribution width-standard deviation; RDW-CV – red cell distribution width-coefficient of variation; SEM – standard error of the mean; bold black text indicates mean values above laboratory's references, whereas bold blue text indicates mean values below laboratory's references

Table S2. Hepcidin, IL-6, TNF-α, CRP, and SAA_1 concentrations in non-anaemia and anaemia women who had one complete blood count examination (upon hospital admission).

	Anaemia upon admission to the hospital	
**Parameter**	**No-anaemia (N=3)**	**Anaemia (N=1)**
Hepcidin [ng/mL]		
Mean ± SEM	80.02 ± 34.16	194.56
Median	109.16	
Min – Max	11.94 – 118.97	
IL-6 [pg/mL]		
Mean ± SEM	8.74 ± 1.56	126.81
Median	7.26	
Min – Max	7.11 – 11.86	
TNF-α [pg/mL]		
Mean ± SEM	2.09 ± 0.73	302.38
Median	1.38	
Min – Max	1.35 – 3.55	
CRP [mg/L]		
Mean ± SEM	9.57 ± 6.77	2.78
Median	5.28	
Min – Max	0.60 – 22.84	
SAA_1 [µg/mL]		
Mean ± SEM	131.07 ± 71.62	81.11
Median	131.07	
Min – Max	59.45 – 202.68	

IL-6 – interleukin 6; TNF-α - tumour necrosis factor α; CRP – C-reactive protein; SAA_1 – serum amyloid A_1; SEM – standard error of the mean

Table S3. Hepcidin, IL-6, TNF-α, CRP, and SAA_1 concentrations in non-anaemia women and women with post-operative anaemia who had two complete blood counts.

	Post-operative anaemia in the day of surgery	
**Parameter**	**No-anaemia (N=21)**	**Developed anaemia (N=2)**
Hepcidin [ng/mL]		
Mean ± SEM	106.33 ± 15.30	49.75 ± 16.41
Median	91.33	49.75
Min – Max	15.20 – 239.36	33.34 – 66.16
IL-6 [pg/mL]		
Mean ± SEM	26.42 ± 3.69	31.54 ± 26.08
Median	24.39	31.54
Min – Max	0.32 – 57.28	5.46 – 57.61
TNF-α [pg/mL]		
Mean ± SEM	2.27 ± 0.24	2.14 ± 0.43
Median	2.06	2.14
Min – Max	1.21 – 5.54	1.71 – 2.56
CRP [mg/L]		
Mean ± SEM	8.04 ± 4.12	10.92 ± 10.32
Median	2.13	10.92
Min – Max	0.25 – 84.72	0.60 – 21.23
SAA_1 [μg/mL]		
Mean ± SEM	81.59 ± 23.51	134.83 ± 3.53
Median	7.33	134.83
Min – Max	0.21 – 330.01	131.30 – 138.35

IL-6 – interleukin 6; TNF-α - tumour necrosis factor α; CRP – C-reactive protein; SAA_1 – serum amyloid A_1; SEM – standard error of the mean

Table S4. Hepcidin, IL-6, and TNF-α concentrations in (a) males with anaemia since hospital admission without and with comorbidities, (b) males who developed anaemia during hospitalization without and with comorbidities, and (c) males with no anaemia during hospitalization without and with comorbidities

**(a)**	Males with anaemia since hospital admission	
**Parameter**	**Without comorbidities**	**With comorbidities**
Hepcidin [ng/mL]		
Mean ± SEM	156.77 ± 33.35	177.44 ± 34.21
Median	180.89	144.02
Min – Max	0.08 – 380.71	1.58 – 525.15
IL-6 [pg/mL]		
Mean ± SEM	21.92 ± 5.32[a]	46.33 ± 5.61[a]
Median	22.24	45.87
Min – Max	0.36 – 59.84	3.97 – 112.70
TNF-α [pg/mL]		
Mean ± SEM	4.05 ± 1.69	2.68 ± 0.41
Median	2.38	2.21
Min – Max	0.67 – 20.57	0.57 – 8.92
**(b)**	Males who developed anaemia during their hospitalization	
	**Without comorbidities**	**With comorbidities**
Hepcidin [ng/mL]		
Mean ± SEM	234.75 ± 82.20	161.41 ± 33.78
Median	259.62	115.17

Min – Max	81.59 – 363.05	39.16 – 429.52
IL-6 [pg/mL]		
Mean ± SEM	28.31 ± 5.78	46.81 ± 11.42
Median	27.42	46.14
Min – Max	18.77 – 38.74	0.21 – 113.83
TNF-α [pg/mL]		
Mean ± SEM	2.43 ± 0.54	11.19 ± 8.40
Median	2.43	1.92
Min – Max	1.89 – 2.96	1.29 – 111.55
**(c)**	Males with no anaemia during their hospitalization	
	**Without comorbidities**	**With comorbidities**
Hepcidin [ng/mL]		
Mean ± SEM	203.60 ± 22.96	141.55 ± 23.83
Median	169.25	143.16
Min – Max	128.42 – 353.74	31.44 – 247.96
IL-6 [pg/mL]		
Mean ± SEM	32.50 ± 9.73	21.50 ± 3.78
Median	21.60	26.13
Min – Max	5.26 – 100.72	5.80 – 37.00
TNF-α [pg/mL]		
Mean ± SEM	2.86 ± 0.59	4.42 ± 1.07
Median	1.93	3.01
Min – Max	1.30 – 7.61	1.46 – 10.95

IL-6 – interleukin 6; TNF-α - tumour necrosis factor α; SEM – standard error of the mean; Statistical significances, Student's t-test: [a]p=0.005271 in IL-6 concentrations between males with anaemia since hospital admission and without comorbidities, and males with anaemia since hospital admission and with comorbidities

Table S5. Hepcidin, IL-6, TNF-α, CRP, and SAA_1 concentrations in non-anaemia and anaemia men who had one complete blood count examination (upon hospital admission).

	Anaemia upon admission to the hospital	
**Parameter**	**No-anaemia (N=5)**	**Anaemia (N=2)**
Hepcidin [ng/mL]		
Mean ± SEM	155.97 ± 49.01	82.09 ± 3.84
Median	154.87	82.09
Min – Max	31.44 – 295.55	78.25 – 85.92
IL-6 [pg/mL]		
Mean ± SEM	12.91 ± 2.72	15.59 ± 7.56
Median	10.60	15.59
Min – Max	9.80 – 18.33	8.03 – 23.15
TNF-α [pg/mL]		
Mean ± SEM	2.69 ± 0.41	3.19 ± 1.25
Median	2.78	3.19
Min – Max	1.71 – 4.01	1.94 – 4.44
CRP [mg/L]		
Mean ± SEM	12.01 ± 8.70	65.81 ± 15.60
Median	4.40	65.81
Min – Max	0.60 – 46.46	50.21 – 81.40
SAA_1 [μg/mL]		
Mean ± SEM	61.54 ± 57.38	150.90 ± 47.32
Median	5.19	150.90
Min – Max	1.26 – 291.01	103.58 – 198.21

IL-6 – interleukin 6; TNF-α - tumour necrosis factor α; CRP – C-reactive protein; SAA_1 – serum amyloid A_1; SEM – standard error of the mean

Table S6. Descriptive statistics of complete blood count parameters from the first and second complete blood count examinations, as well as hepcidin, IL-6, TNF-α, CRP, and SAA_1 levels in men with and without anaemia and men with post-operative anaemia.

	The prevalence of anaemia in the day of surgery (after surgery)					
	No-anaemia (N=15)		Developed anaemia (N=7)		Anaemia (N=16)	
Parameter	**1st CBC**	**2nd CBC**	**1st CBC**	**2nd CBC**	**1st CBC**	**2nd CBC**
NCI grading [n (%)]						
Grade 0	15 (100%)	15 (100%)	7 (100%)	-	-	-
Grade 1	-	-	-	7 (100%)	16 (100%)	12 (75%)
Grade 2	-	-	-	-	-	4 (25%)
Grade 3	-	-	-	-	-	-
Grade 4	-	-	-	-	-	-
Hb [g/dL]						
Mean ± SEM	14.82 ± 0.18[a,b]	14.30 ± 0.16	13.96 ± 0.15[a,c]	12.70 ± 0.24	11.83 ± 0.27[b,c]	11.28 ± 0.34
Median	14.80	14.20	13.80	12.80	11.80	11.05
Min – Max	13.80 – 16.60	13.50 – 15.40	13.50 – 14.60	11.40 – 13.30	10.00 – 13.40	9.40 – 13.80
RBC [mln/μL]						
Mean ± SEM	4.75 ± 0.08	4.58 ± 0.09	4.57 ± 0.10	4.26 ± 0.15	3.99 ± 0.15	3.78 ± 0.14
Median	4.79	4.66	4.61	4.27	4.06	3.69
Min – Max	4.23 – 5.24	4.00 – 5.17	4.25 – 4.91	3.70 – 4.92	2.83 – 5.27	2.94 – 4.63
Hepcidin [ng/mL]						
Mean ± SEM	161.50 ± 12.41[d]		90.84 ± 21.93[d]		126.50 ± 33.29	
Median	165.90		81.59		140.67	
Min – Max	85.39 – 243.65		39.16 – 205.37		0.08 – 344.26	
IL-6 [pg/mL]						
Mean ± SEM	21.34 ± 3.69		25.93 ± 9.93		29.08 ± 5.52	
Median	24.86		18.77		34.37	
Min – Max	5.26 – 46.68		10.56 – 64.75		2.63 – 58.34	
TNF-α [pg/mL]						
Mean ± SEM	4.12 ± 0.85		19.23 ± 15.45		3.60 ± 1.31	
Median	2.41		2.96		1.91	
Min – Max	1.30 – 10.95		1.29 – 111.55		0.57 – 20.57	
CRP [mg/L]						
Mean ± SEM	21.58 ± 8.52		19.98 ± 12.53		32.79 ± 12.25	
Median	1.22		7.87		12.35	
Min – Max	0.22 – 106.44		1.08 – 93.34		0.60 – 153.93	
SAA_1 [μg/mL]						
Mean ± SEM	101.86 ± 38.96		105.15 ± 53.91		119.10 ± 30.52	
Median	10.96		19.83		127.67	
Min – Max	0.62 – 548.31		2.63 – 336.87		0.75 – 395.53	

CBC – complete blood count; NCI – National Cancer Institute; Hb – haemoglobin; RBC – red blood count; IL-6 – interleukin 6; TNF-α - tumour necrosis factor α; CRP – C-reactive protein; SAA_1 – serum amyloid A_1; SEM – standard error of the mean; Statistical significances, One-way ANOVA: [a]p=0.023919 in haemoglobin concentration upon hospital admission between men who did not have anaemia and men who developed anaemia post-operatively; [b]p=0.000123 in haemoglobin concentration upon hospital admission between men who did not have anaemia and men who had anaemia; [c]p=0.000123 in haemoglobin concentration upon hospital admission between men who developed anaemia post-operatively and men who had anaemia; [d]p=0.007905 in hepcidin concentration between men without anaemia and men that developed anaemia post-operatively
